# Gut microbiome in non-alcoholic fatty liver disease

**DOI:** 10.3389/fgstr.2024.1534431

**Published:** 2025-01-14

**Authors:** Anastasios Mpountouridis, Christina Tsigalou, Ioanna Bezirtzoglou, Eugenia Bezirtzoglou, Elisavet Stavropoulou

**Affiliations:** ^1^ Gastroenterology Department, Theagenio Cancer Hospital, Thessaloniki, Greece; ^2^ Laboratory of Hygiene and Environmental Protection, Faculty of Medicine, Democritus University of Thrace, Alexandroupolis, Greece; ^3^ School of Chemistry, University of Edinburgh, Scotland, United Kingdom

**Keywords:** non-alcoholic fatty liver disease (NAFLD), non-alcoholic steatohepatitis (NASH), gut microbiome, gut microbiota, gut dysbiosis

## Abstract

Non-alcoholic fatty liver disease (NAFLD) has a rapidly growing incidence worldwide, affecting approximately one-third of world population. The disturbance of gut commensal bacteria impacting host’s homeostasis is referred to as gut dysbiosis. The gut microbiome contributes to the pathogenesis of NAFLD through various pathways. Gut microbiota is at constant interactions with the intestinal epithelial barrier and affects its integrity. Through gut-liver axis, gut microbiota may influence liver immune function. The release of lipopolysaccharides (LPS) from intestines to portal vein which are transported to the liver, may trigger hepatic inflammation, steatosis and even fibrosis. Moreover, the gut microbiome induces the conversion of primary bile acids (BAs) to secondary BAs, which activates intestinal receptors, such as FXR and TGR5. FXR activation decreases fat absorption and thus reduces hepatic lipid accumulation, while TGR5 activation promotes the release of glucagon-like peptide-1 (GLP-1) in blood. Furthermore, gut ethanol-producing bacteria has been implicated in NAFLD development. Additionally, in NAFLD there is a reduction in intestinal levels of short-chain fatty acids, such as butyrate, propionate and acetate. Many bacterial alterations have been observed in NAFLD, including the increased *Bacteroidetes* and decreased *Firmicutes*. Many probiotics have been tried in NAFLD prevention and management, including a plethora of strains from *Lactobacilli*, *Bifidobacteria* and *Streptococcus* and some of them have promising perspectives. There is also some promising data from the administration of prebiotics (such as inulin and fructo-oligosaccharides) and symbiotics (probiotics plus prebiotics). Faecal microbiota transplantation (FMT) is yet to be evaluated for its efficacy against NAFLD.

## Introduction

Non-alcoholic fatty liver disease (NAFLD) is a liver disease defined by the accumulation of more than 5% of fat in the liver as triglycerides inside the hepatocytes (1). NAFLD includes a wide spectrum of diseases that range from simple liver steatosis without inflammation to more severe conditions such as non-alcoholic steatohepatitis (NASH), which is also known as metabolic dysfunction-associated steatohepatitis (MASH), characterised by inflammation, which causes varying degrees of liver fibrosis and even cirrhosis (2). This disease affects approximately one-third of the population worldwide, and its incidence is higher in men than women (3). The vast majority of the affected patients may not experience any symptoms until the disease progresses to more advanced stages, making NAFLD a potential silent killer shortly (4). Moreover, NAFLD is related to many metabolic disorders such as diabetes mellitus type 2, central obesity, hypertension, dyslipidemia and abnormal liver function tests (5). NAFLD is a common cause of chronic liver disease and cirrhosis, among other aetiologies such as alcohol consumption and viral hepatitis, while cirrhosis caused by NAFLD has considerably increased worldwide over the past decades (6). Moreover, there are many reports of hepatocellular carcinoma in NAFLD, and some of them occurred even without the presence of cirrhosis (7).

Gut microbiota is defined as the microbial community that resides inside the whole gastrointestinal tract, while the microbiome refers to the genetic information within the microbiota, including bacteria, fungi and viruses, all of which exist on and in the human body, which is the host (8). The human intestinal tract is colonised with approximately 3,8 x 10^13^ bacteria, whereas the human cells account for roughly 3 x 10^13^ (9). Furthermore, gut microbiome sequences contain 3,3 x 10^6^ microbial genes, a huge number that is 150 times bigger than the human genes, which are 2,2 x 10^4^ (10). Microbiome has a crucial role in the development of the host’s immune system (11). Parallelly, gut microbiome disturbance is associated with a plethora of diseases, such as cardiovascular (12), autoimmune and inflammatory diseases (13), and it is affiliated with various types of cancer (14). In addition, the gut microbiome’s diversity has an impact on neurological and psychiatric disorders, like Alzheimer’s disease and depression, respectively, via the gut-brain axis (15, 16). The type of childbirth and the complementary feeding transition during the first year of life are critical parameters in the acquisition and development of the infant microbiome (17, 18). Nevertheless, constantly throughout the whole lifespan of the host, the gut microbiome remains susceptible to alterations due to many factors such as lots of medications, dietary habits and preferences, health status, stress, various environmental agents and ageing (19).

## The role of gut microbiome in intestinal function

Most cells of the immune system are located in the intestinal lumen, containing innate lymphoid cells, γδ T cells, type 1 interferon-producing plasmacytoid dendritic cells and mucosa-associated invariant T cells (20). The intestinal epithelial barrier, established at the luminal surface, consists of physical, microbial and immunologic constituents, compromising tight intracellular junctions between intestinal epithelial cells (21). The gut microbiome and intestinal epithelial barrier are in continuous dynamic interaction with the immune cells, epithelial cells and the gut microbiome being the protagonists in this beneficial coexistence (22). A secure and regulated intestinal barrier has an important role in preventing the translocation of bacteria outside of the intestinal lumen (23). Commensal bacteria reinforce intestinal barrier integrity via Toll-like receptors (TLR) signalling, inducing gut epithelial cell proliferation as well as stimulation of cell-mediated immunity (24). By secreting immunoglobulin IgA and antimicrobial peptides such as α-defensins, β-defensins, lysozyme, C-type lectins and cathelicidins, the immune system contributes to the integrity of the intestinal barrier (25). However, a potential disturbance in the commensal gut microbial communities can cause intestinal disease by the activation of the immune system and malfunction of the intestinal barrier and, thus, harmful intestinal permeability (26). This condition is known as gut dysbiosis, and it is related to many diseases, either intestinal or systemic, such as inflammatory bowel disease (IBD) (27), irritable bowel syndrome (IBS) (28), diabetes mellitus type 2 (29), etc. In addition, intestinal permeability, which refers to the disturbance of tight intercellular junctions, has been linked to NAFLD occurrence (30).

## Gut microbiome and hepatic inflammation

**Figure 1:**
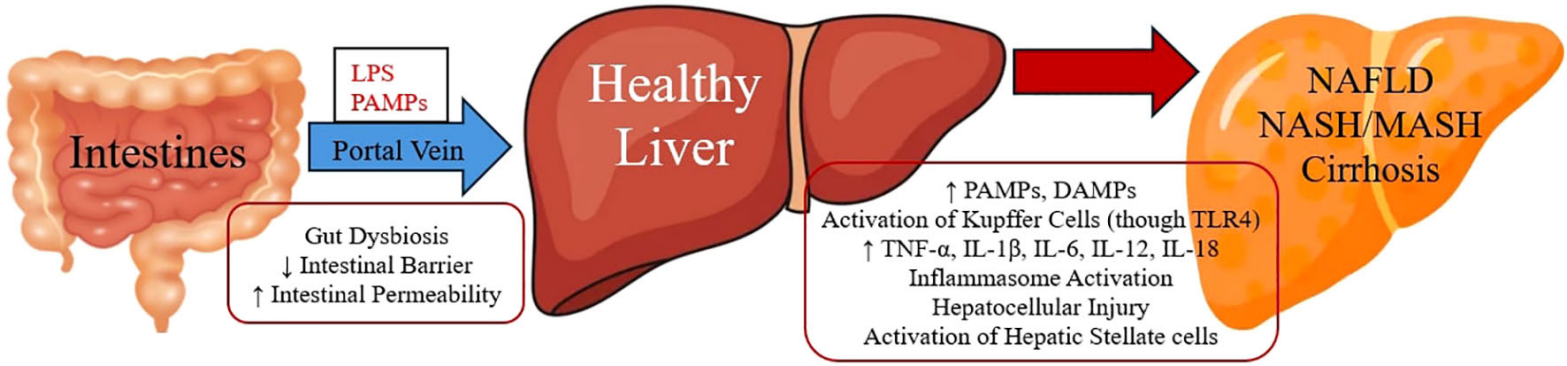
Gut Microbiome and Liver Inflammation.

The liver and gut are in constant interaction through the gut-liver axis, which refers to the physical connection via the portal vein and blood inflow directed to the liver from the intestines (31). Growing investigational data supports the theory of intestinal barrier dysfunction blamed for triggering inflammation in the liver tissue, leading to NAFLD progression (32). Conversely, restoration of intestinal barrier function may have an alleviative impact on liver inflammation and thus a mitigation to NAFLD and fibrosis development (33). Disturbance of the intestinal barrier and, thus, intestinal permeability permits the entrance of gut microorganisms into the bloodstream, and via the portal vein, they direct at the liver, provoking hepatocellular injury through activation of immune cells (34). This phenomenon of bacteria and their membrane molecules, such as lipopolysaccharides (LPS), directed to the liver through portal vein is also known as translocation (35). Inside the liver, Kupffer cells (macrophage cells) with their toll-like receptor 4 (TLR4) can recognise LPS and other pathogen associated microbial patterns (PAMPs) that are components of the bacteria and activate further inflammatory cells (including neutrophils, monocytes and T lymphocytes) directed to liver by expressing cytokines (such as TNF-α, IL-1β, IL-6, IL-12, IL-18) and other signalling molecules (36). This procedure can lead to a more severe form of NAFLD, namely non-alcoholic steatohepatitis (NASH), which is also known as metabolic dysfunction-associated steatohepatitis (MASH), evoking inflammation with hepatocellular injury, which may cause different degrees of fibrosis by the activation of hepatic stellate cells, and this can lead even to liver cirrhosis (35, 37). Vice versa, it is observed that animal models lacking TLR4 receptors are not capable of binding LPS, and thus, they are protected from hepatic steatosis development (38). Moreover, damaged parenchymal and nonparenchymal cells from intestines and liver are able to release damage-associated molecular patterns (DAMPs), which as well as PAMPs can trigger hepatic innate immune cells through TLRs activation (39). Both DAMPs and PAMPs (such as LPS) can activate inflammasome sensors that can stimulate the intracellular increase of multiprotein complex as the effector protein caspase-1, which can lead to IL-1β and IL-18 release and stimulation of the pyroptotic protein gasdermin D (GSDMD), provoking hepatic cells death (40). In NAFLD patients, elevated serum levels of IL-1β and IL-18 have been observed as well as increased NLRP3 (NLR family pyrin domain containing 3) inflammasome activation, inducing hepatocytes death (41) (**Figure 1**).

## Metabolism of bile, enterohepatic circulation and gut microbiome

The primary bile acids (BAs), chenodeoxycholic acid and cholic acid, are synthesised in the liver, and then through conjugation with either taurine or glycine, they form bile salts as a component of bile, which is stored in the gallbladder and through the biliary system it is secreted into the intestinal lumen (42). In the gut, bile salt hydrolases (BSHs) deconjugate BAs from bile, while BSHs have been found in gut microbial *phyla*, such as *Bifidobacterium*, *Bacteroides*, and microbial *genera*, including *Lactobacillus*, *Clostridium* spp. and *Enterococcus* (43). Afterwards, inside the intestine, they are metabolised into diverse secondary bile acids (deoxycholic acid from cholic acid and lithocholic acid from chenodeoxycholic acid) by gut microbiota, with the enzyme 7alpha-dehydroxylase, which is mostly synthesised by species from the *Firmicutes phylum* (43–45). The vast majority (95%) of all bile acids are actively reabsorbed in the last part of the small intestine (Ileum), and through the portal vein, they return to the liver, where they are reused many times daily (46).

**Figure 2:**
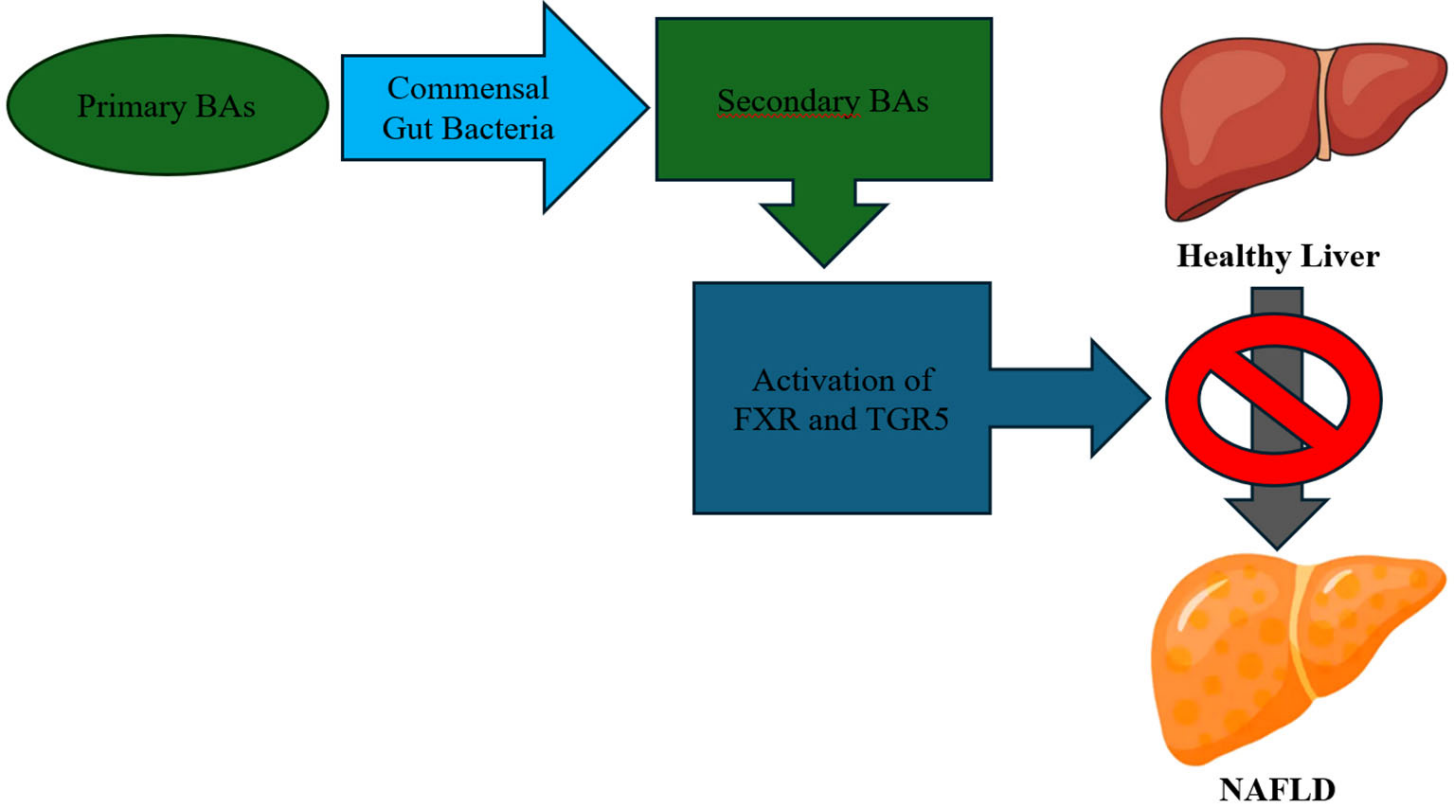
Bile Acids (BAs) – Mechanism of Action.

Secondary BAs are crucial for the absorption of lipids and other nutrients from the intestinal tract (42). Moreover, secondary BAs have a ligand role in numerous receptors, such as the nuclear farnesoid X receptor (FXR), regulating bile synthesis and metabolism (47). In addition, BAs may activate or modulate bile acid nuclear receptors, such as FXR, PXR and TGR5, a vitamin D receptor (NR1I1), and transporters, such as the ileal apical sodium-dependent bile acid transporter (ASBT), some of which seem to contribute to NAFLD and NASH pathogenesis and progression, as well as they have an impact on insulin resistance (48). In experimental models, it has been found that activation of FXR by agonist agents reduces lipid absorption and decreases the accumulation of monosaturated and polyunsaturated fatty acids in the liver (49). In a clinical trial with NASH patients, Rinella ME et al. (2022) observed improvements in non-invasive liver tests in the obeticholic acid (FXR agonist agent) group to the placebo group (50). Activation of TGR5 receptor promotes the increase of glucagon-like peptide-1 (GLP-1) levels in blood (51). Administration of GLP-1 receptor agonists to diabetic patients has been linked with decreased fatty liver, hyperlipidemia and hypertension (52). Furthermore, in experimental models, TGR5 receptor activation downregulates (NF-kappaB)-mediated inflammation and thus, it has an anti-inflammatory effect on the liver in rodents (53) (**Figure 2**). Experimental inhibition of ASBT transporter has shown a reduction in body weight, intestinal fat absorption and hepatic steatosis in mice, but further research is needed (54).

When commensal diverse gut microbiota is disrupted, there is a decrease in converting primary to secondary BAs and thus reduced activation of bile acid receptors. Also, decreased secondary BAs evoke further disturbance to bacterial symbiosis (55). Via FXR signalling, BAs can protect commensal gut microbiota from gut bacteria overgrowth and reinforce intestinal epithelial barrier (56). In NAFLD, there is a reduction in gut bacteria that convert primary into secondary BAs (57). It has been found that the increased ratio of conjugated chenodeoxycholic acid (CDCA)/muricholic acid (MCA) in serum was related to worse NASH progression in 134 individuals having NAFLD (58). Furthermore, in FXR lacking rodents, a reduction in deconjugation of bile salts decreases the release of taurine, which has a beneficial effect on hepatic inflammation and steatosis (59). Ursodeoxycholic acid (UDCA) belongs to secondary BAs, which can be administrated orally as a medication, and with its primary colonic metabolite, lithocholic acid, they can have anti-inflammatory effects on the colon (60). In an experiment with mice, co-administration of UDCA with a statin (rosuvastatin) and ezetimibe decreased the accumulation of collagen in rodents’ liver and ALT (alanine aminotransferase) levels in serum and improved fibrosis-related markers, seeming to be a promising therapy in NAFLD deterioration, but further investigation is needed (61). Furthermore, external factors, such as dietary habits and medications, may influence the bile acid pool and its actions indirectly through their effect on the gut microbiome (62).

## Ethanol-producing bacteria in NAFLD

The characteristics of liver steatosis and inflammation are very similar in alcoholic fatty liver disease and in non-alcoholic fatty liver disease (NAFLD) (63). The gut microbial community can produce ethanol, which is absorbed from the intestine and through the portal vein, which proceeds to the liver (64, 65). Baker SS, et al. (2010) found an increased ADH (alcohol dehydrogenase) gene transcription in the NASH group compared to the control group, suggesting increased blood alcohol levels in NASH patients and increased activity of metabolising the circulating alcohol in NASH livers (65). Ethanol-producing gut microbiota is one of the multiple factors and mechanisms of the progression of NAFLD and deterioration to NASH (66). In NASH subjects, an increased proportion of alcohol-producing bacteria has been found in their gut, as well as increased ethanol blood levels (67).

From the gut bacteria, a high-alcohol-producing species of *Klebsiella pneumoniae* was associated with NAFLD in humans, and when this species was transferred into rodents’ intestines, it provoked NAFLD as well (68). Furthermore, Mbaye B, et al. (2023) observed increased ethanol and glucose in the faeces of NASH individuals, which was related to dysbiosis and alteration of gut microbiome with augmentation of ethanol-producing bacteria, such as *Enterocloster bolteae*, *Limosilactobacillus fermentum*, *Streptococcus mutans* and *Mediterraneibacter gnavus* (69). In the liver, alcohol can provoke mitochondrial dysfunction with pathological fatty acid oxidation and impaired oxidative phosphorylation, causing oxidative stress in hepatocytes, which can lead to steatohepatitis (70). Meijnikman AS, et al. (2022) observed median ethanol concentrations in the portal vein were 187 times higher compared to fasting peripheral blood. Also, ethanol levels were increased proportionally from individuals without liver steatosis to NAFLD and even increased in NASH (71). In the same study, applying inhibition of ADH (a liver enzyme which metabolises ethanol) in NAFLD individuals increased 15 times ethanol concentrations in peripheral blood, but this phenomenon was ameliorated after the administration of antibiotics (71). Ethanol-producing bacteria, among many others, play a role in the pathogenesis of NAFLD, and the gut microbiome might be one of the potential therapeutic targets of this disease (72).

## The role of short-chain fatty acids

Short chain fatty acids (SCFAs) are produced inside the intestinal lumen by the gut commensal bacteria during the fermentation of dietary fibres, with the main SCFAs being butyrate, propionate and acetate (73). Inadequate fibre consumption may compromise the production of SCFAs, impairing the host’s immune system function, which may be associated with variant diseases (74). SCFAs improve the function of epithelial intestinal cells by regulating their proliferation and differentiation, providing energy to gut epithelial cells, enhancing the host’s metabolism and reinforcing the epithelial gut barrier (75). A high fat/carbohydrate diet may promote dysbiosis in gut microbiota by the predominance of *Prevotella*, *Firmicutes* (*Clostridium*) and *Methanobrevibacter*, diminishing the beneficial bacteria (*Bifidobacterium*, *Bacteroides*, *Akkermansia* and *Lactobacillus*), which is related to reduced SCFAs production, increased inflammation, dyslipidemia and obesity (76). Butyrate has the most anti-inflammatory properties by activating T-regulating immune cells, which act with the inhibition of T cells and Th17, intervening in the inflammatory cascade (77).

Alteration of gut microbiota may impair the SCFAs production, which may cause obesity-related diseases, including NAFLD (78). Decreased levels of butyrate are linked to intestinal barrier dysfunction, intestinal permeability, and translocation of bacterial endotoxins (LPS), causing liver steatosis (79). Zhou D, et al. (2017) administrate sodium butyrate to high-fat diet rodents, which resulted in the restoration of gut microbiota dysbiosis, improvement of gut barrier function and amelioration of inflammation and fat accumulation in the liver (80). Furthermore, SCFAs, through the activation of free fatty acid receptors in the intestine, release hormones, such as glucagon-like peptide-1 (GLP-1) and peptide YY, that regulate the host’s glucose levels, appetite and energy metabolism (81). In high-fat diet animal models, sodium butyrate can upregulate intestinal L cells to release GLP-1, which activates the GLP-1 receptor with a beneficial effect on the progression of NAFLD and NASH, while butyrate can also upregulate the expression of GLP-1 receptors in the liver (82). Moreover, sodium butyrate seems to attenuate the deterioration of NAFLD by reducing the inflammation in the liver and protecting melatonin production and receptor expression in the liver and small intestine (83).

## Endocannabinoid system and NAFLD

Endocannabinoid system seems to play a noteworthy role in the regulation of lipid, glucose and energy metabolism, as well as in immune function and inflammation (84). Obesogenic high‐fat diets may affect microbiota-gut-brain axis by increasing endocannabinoid levels in peripheral tissues and brain (85). Furthermore, the endocannabinoid system, among other metabolic systems, regulates the intestinal barrier function (86). It has been found that pharmacological activation of endocannabinoid system may lessen intestinal barrier integrity and provoke adipogenesis (87). Gut microbiota is able to produce variant metabolites, including endocannabinoids, regulating the development of adipose tissue and its metabolic function (88). Agonists of cannabinoid receptors (type 1 and 2), like bioactive lipids from N-acylethanolamine family, may promote metabolic disorders and hepatic steatosis (89). Disrupted endocannabinoid system regulation and tissue metabolism have been found in obese and diabetic mice with altered gut microbiota (90). Moreover, changes in intestinal and plasma endocannabinoid levels may cause modifications in hypothalamic Pomc neurons’ function, inducing hyperphagic behaviour and exacerbating obesity and hepatic steatosis in rodents (91).

## The role of choline in NAFLD

Choline is an essential nutrient for normal liver function by packaging and exporting triglycerides in very low-density lipoprotein (VLDL) from hepatic tissue, while low-choline diet is associated with NAFLD (92). In high-fat diet pig model, gut microbiota may catabolise choline to trimethylamine that is converted to trimethylamine-N-oxide which can accumulate into liver and, along with choline depletion, may induce NASH (93). The metabolism of choline by gut microbiota regulates the bioavailability of choline from the diet (94). Moreover, higher dietary choline is related with lower risk of developing NAFLD compared to inadequate choline consumption in both males and females (95). Furthermore, methionine-choline-deficient-diet has been used in many animal studies to provoke NAFLD models (54, 96, 97).

## Gut microbial alterations and NAFLD

It has been observed in a plethora of studies that there is a tight relationship between intestinal dysbiosis and NAFLD (98). Wigg AJ, et al. (2001) found increased small intestinal bacterial overgrowth (SIBO) prevalence in NASH to control individuals (assessed by breath test with ingestion (14)C-D-xylose-lactulose), as well as higher tumour necrosis factor alpha levels in NASH group, proposing the contribution of SIBO in NASH pathogenesis (99). Zhang X, et al. (2020) observed in mice that a high-cholesterol diet can provoke gut microbiota dysbiosis by increasing some *species* (*Anaerotruncus*, *Desulfovibrionaceae*, *Desulfovibrio* and *Mucispirillum*) while decreasing others (*Bacteroides* and *Bifidobacterium*), inducing the development and deterioration of NAFLD (100). In stool samples, Mouzaki M, et al. (2013) found a decreased percentage of *Prevotella species* (*Bacteroides*) in the NASH group in comparison with the simple steatosis group and healthy liver group, regardless of BMI and dietary fat intake (101). The prevalence level of SIBO is higher in NASH in healthy individuals, inducing inflammation and fibrosis in the liver through the increased levels of endotoxins, which activate the immune system with upregulation of toll-like receptor 4 (TLR-4) expression and releasing the pro-inflammatory cytokine, interleukin 8 (IL-8) (102).

In NAFLD, there is a reduced diversity in gut microbiota, and as for the *phyla*, compared to healthy controls, the NAFLD individuals have increased *Bacteroidetes* and decreased *Firmicutes* in their faecal samples (103). Furthermore, increased gut *Proteobacteria* has been observed in obese non-diabetic women with hepatic steatosis (104). In addition to *Proteobacteria*, *Fusobacteria phyla* are also increased in NALFD and regarding gut bacterial *families* in NAFLD, there is an augmentation in *Enterobacteriaceae* and *Lachnospiraceae*, while the *Ruminococcaceae* and *Prevotellaceae families* are depleted compared to healthy individuals (105). Moreover, decreased *Oscillospira* and *Rikenellaceae families* have been found in NALFD, while increased *Dorea genus* in the gut microbiome relates to the deterioration of NAFLD to NASH (57). Additionally, increased *Escherichia genus* and *Peptoniphilus genus* have been observed in NAFLD patients (57, 67). On the contrary, a reduction is present in the *genera* of *Faecalibacterium* (67), *Coprococcus* (103), *Anaerosporobacter* (103), *Eubacterium* (102) and *Prevotella* (106) in NAFLD gut microbiota samples (**Table 1**). The former gut microbiota imbalance and gut dysbiosis have promoted the introduction of microbiome-target therapies, including prebiotics, probiotics, synbiotics (probiotics plus prebiotics) and faecal microbiota transplantation (FMT) (107).

**Table 1:** Gut microbiota alterations in NAFLD.

Study Author and Year	GUT Microbiota findings in NAFLD
Zhang x, et al. (2020) (100)	↑ *Anaerotruncus*, *Desulfovibrionaceae*, *Desulfovibrio* and *Mucispirillum* s*pecies* ↓ *Bacteroides* and *Bifidobacterium* s*pecies*
Mouzaki m, et al. (2013) (101)	↓ *Prevotella species* (*Bacteroides*)
Shanab aa, et al. (2011) (102) and Wigg aj, et al. (2001) (99)	↑ Percentage of small intestinal bacterial overgrowth (SIBO)
Wang b, et al. (2016) (103)	↑ Gram-negative bacteria ↑ *Bacteroidetes phylum* ↓ *Firmicutes phylum* ↓ *Coprococcus* and *Anaerosporobacter genera* ↓ Microbiota diversity
Hoyles l, et al. (2018) (104)	↑ *Proteobacteria phylum* ↓ *Eubacterium genus*
Shen f, et al. (2017) (105)	↑ *Proteobacteria* and *Fusobacteria phyla* ↑ *Enterobacteriaceae*, *Lachnospiraceae*, *Erysipelotrichaceae* and *Streptococcaceae families* *↑ Escherichia_Shigella*, *Lachnospiraceae_Incertae_Sedis* and *Blautia genera* ↓ *Bacteroidetes phylum* ↓ *Ruminococcaceae* and *Prevotellaceae families*
Del chierico f, et al. (2017) (57)	↑ *Actinobacteria phylum* ↑ *Bradyrhizobium*, *Anaerococcus*, *Dorea*, *Escherichia*, *Propionibacterium acnes*, *Ruminococcus* and *Peptoniphilus genera* ↓ *Bacteroidetes phylum* ↓ *Oscillospira* and *Rikenellaceae families* ↓ Microbiota Diversity
Zhu l, et al. (2013) (67)	↑ Alcohol-producing bacteria ↑ *Proteobacteria phylum* ↑ *Escherichia*, *Prevotella* and *Peptoniphilus genera* ↓ *Faecalibacterium genus*
Boursier j, et al. (2016) (106)	↑ *Bacteroides genus* ↓ *Prevotella genus*

↑: increased.

↓: decreased.

## Probiotics in NAFLD

The definition of probiotics is live microorganisms that, when administered in adequate amounts, confer a health benefit on the host (108). Administration of probiotics may be a potent therapy in NAFLD with a decrease in liver fat accumulation and restoration of liver aminotransferases blood levels (109). Probiotics can modulate gut microbiota, enhance intestinal barrier, improve hepatic and serum lipid profiles, reduce liver steatosis and have anti-inflammatory effects (110). There are variant probiotic bacteria that have been administered experimentally in rodents with NAFLD with beneficial results, including *Lactobacilli*, *Bifidobacteria* and *Streptococcus* (111). Naudin CR, et al. (2020) noticed that supplementation of *Lactococcus lactis Subspecies cremoris* to western-style (high-fat and/or high-carbohydrate) diet female mice mitigates hepatic inflammation and ameliorates liver steatosis (112). Okubo H, et al. (2013) observed that the administration of *Lactobacillus casei strain Shirota* protects from the development of NASH in methionine-choline-deficient-diet mice through augmentation of gut lactic acid bacteria (*Bifidobacterium* and *Lactobacillus*) (96). In a different animal model, *Lactobacillus casei strain Shirota* prevented the development of NAFLD in fructose-induced steatosis mice by downregulation of Toll-like receptor 4 (TLR4) activity and upregulation of peroxisome proliferator-activated receptor γ (PPAR-γ) activation (113). Additionally, administration of *Bifidobacterium Pseudocatenulatum CECT 7765*, beyond metabolic amelioration, improves immune function in obese high-fat-diet mice, decreasing interleukin six levels and enhancing dendritic and macrophage cells signalling and functioning (114).

Zhao et al. (2020) reported the administration of *Lactobacillus plantarum NA136* led to improvement of NALFD in high-fat and fructose diet mice at various levels, including correction of gut microbiota disturbances, reinforcement of intestinal barrier and decrease of liver inflammation (115). Furthermore, *Lactobacillus plantarum strains* reduced fat accumulation in histopathological examination and improved blood biochemical liver markers in NAFLD rodents, according to Park EJ, et al. (2020) (116). The probiotic *Akkermansia muciniphila* mitigates immune-induced liver histopathological injury triggered by Concanavalin A in a mouse model (117). Administration of *Lactobacillus rhamnosus GG* in high-fructose diet mice with NAFLD enhances gut barrier integrity, decreases lipopolysaccharide levels in the portal vein and lessens the release of pro-inflammatory cytokines, such as TNF-α, IL-8R and IL-1β, from the liver, as well as reduce fat accumulation in liver and ALT-aminotransferase blood levels (118). In addition, *Lactobacillus rhamnosus GG* in high-fat diet obese mice decreases serum cholesterol and triglyceride levels, reduces hepatic fat accumulation, lessens pro-inflammatory and lipogenic gene activation in the liver and downregulates FGF15 and FXR signalling compared to solely high-fat diet obese mice (119). Moreover, on NAFLD mice induced by a high-fat/high-fructose diet plus intermittent hypoxia exposure, *Lactobacillus rhamnosus GG* has a protective effect on insulin resistance, glucose intolerance, hepatic injury and steatosis, and upregulates liver PPARα signalling and increases butyrate faecal levels (120). *Lactobacillus paracasei* ameliorates NASH in mice by inducing the dominance of M2 Kupffer cells and downregulation of M1 Kupffer cells in the liver (121). *Lactobacillus johnsonii BS15* can prevent the development of NAFLD in mice by protecting hepatocytes from oxidative stress and attenuating mitochondrial dysfunction (122). In western-type-diet FXR receptor knockout mice, which develop NASH, administration of probiotic *VSL#3* has a protective effect through activation of alternative bile acid pathway (activation of GPBAR1 receptor), modulation of gut microbiota resulting in increased production of butyrate and thus anti-inflammatory and metabolic benefits (123).

In a double-blind, randomised clinical trial (RCT) with obese children suffering from NAFLD, Alisi, et al. (2014) showed that daily administration of *VSL#3* probiotics (a cluster of 8 distinct lactic acid-producing bacteria) results in amelioration of fatty liver in ultrasonographic examination at 4 months, via upregulation of glucagon-like peptide 1 (GLP-1) signalling (124). Famouri F, et al. (2017) noticed sonographic liver improvement as well as a decrease in aminotransferases blood levels induced by the concurrent administration of 4 probiotic bacteria to obese children having biochemical and sonographic NAFLD (125). Daily consumption of 500 million *Lactobacillus bulgaricus* and *Streptococcus thermophilus* alleviated liver aminotransferases blood levels in patients with NAFLD in a double-blind (2011) RCT (126). Vajro P, et al. (2011) noticed alanine aminotransferase (ALT) reduction after treatment with *Lactobacillus rhamnosus strain GG* in children suffering from obesity-related NAFLD (127). Probiotics can inhibit harmful bacterial proliferation and enhance gut barrier integrity, resulting in a reduction of lipopolysaccharide (LPS) and, thus, downregulation of toll-like receptor four signalling in the liver (128).

A 2023 meta-analysis with 41 RCTs showed that the administration of probiotics, prebiotics or synbiotics can ameliorate sonographic liver steatosis, improve fibrosis and reduce blood levels of aminotransferases (AST and ALT) and gamma-glutamyl transpeptidase (GGT) (129). In a 2019 meta-analysis with 28 clinical trials, including 1555 individuals with NAFLD, probiotics reduce serum levels of aminotransferases (AST and ALT), GGT, total cholesterol and insulin, improve insulin resistance and decrease BMI, while there is no significant impact on lipid profile and TNF-a levels (130). Moreover, a 2021 meta-analysis with 352 patients suffering from NAFLD showed that probiotics can significantly decrease serum levels of aminotransferases (AST and ALT) and total cholesterol, but there is no effect on BMI, insulin resistance and levels of TNF, although there is a reduction in BMI when probiotic treatment surpasses 3 months (131). Either probiotic mixtures or single-strain probiotics can prevent the development of diet-induced NAFLD by restoring gut microbial composition, enhancing gut barrier integrity, inducing fatty acid oxidation and downregulation of lipogenesis in the liver (132). In the elderly, which can potentially have gut microbial alterations, probiotics may restore gut dysbiosis, decrease oxidative stress and thus prevent or mitigate the progression of NAFLD (133).

## Prebiotics in NAFLD

A Prebiotic is defined as a substrate that is selectively utilised by the host’s microorganisms, conferring a health benefit, according to the International Scientific Association for Probiotics and Prebiotics (ISAPP) statement in 2016 (134). Prebiotics are food components which are not digested or absorbed from the intestine, but they are fermented by gut microbiomes, altering the composition of gut microbiota in a favourable way for the host (135). For instance, the consumption of prebiotics, such as galacto-oligosaccharides (GOS), can promote the augmentation of *Bifidobacteria* and *Lactobacilli* inside the intestine (136).

Administration of fructo-oligosaccharides (FOS) to obesity-induced (from injection with monosodium glutamate) mice with NAFLD promotes augmentation of SCFAs production from gut microbiota, and thus reduction of hepatic inflammation and amelioration of steatohepatitis (137). Furthermore, in a high-fat/high-sugar diet mouse model, FOS ameliorates hepatic lipid accumulation, decreases serum levels of total cholesterol, transaminases (ALT and AST) and inflammatory cytokines (IL-6 and TNF-a) and improves lipid profile (138). Consumption of choline and FOS by rodents with NAFLD induces fat degradation in the liver, and parallelly, choline treatment increases the levels of vitamin E and glutathione in hepatic and cardiac tissue (139). In mice which were fed with a methionine-choline-deficient diet, the addition of FOS to their diet prevents the *Lactobacillales* spp. Reduction and *Clostridium cluster XI* augmentation in gut microbiota, increases faecal SCFAs and IgA concentrations, downregulates toll-like receptors 4 (TLR4) function in the liver and mitigates hepatic inflammation and steatosis (97). Furthermore, in a mouse model underlain n-3 PUFA-depleted diet, FOS supplementation induces augmentation of *Bifidobacterium* spp. and reduction in *Roseburia* spp. in the gut, and it mitigates fat accumulation in the liver through PPAR-α genes upregulation (140).

Inulin, as a fructan-type prebiotic, can increase, through fermentation, the production of SCFAs by gut microbiota, increase omega-3 and odd-chain fatty acids levels and downregulate the expression of genes promoting lipogenesis (Fasn, Gpam) in the liver, as observed in rodents (141). In a 2020 randomised, double-blind clinical trial with patients suffering from NAFLD, who underlain weight loss through a very-low-calorie diet, the administration of inulin plus short therapy with metronidazole to these patients decreases alanine aminotransferase (ALT) levels (142). Administration of inulin-type fructans, including oligofructose, to patients with NASH reduces aminotransferases and insulin blood levels (143). Supplementation with inulin to western-type diet obese mice enhances intestinal barrier integrity, decreases endotoxemia and ameliorates hepatic steatosis (79). Moreover, inulin restores gut microbiota disturbances, increases SCFAs (particularly butyrate and propionate) synthesis inside the intestine, increases activation of PPAR-α receptor and mitigates liver inflammation and steatosis in high-sucrose diet rodents with NAFLD (144). In addition, inulin may alleviate NAFLD through augmentation of bile acids synthesis in the liver, increased bile acids excretion to the intestine and upregulation of FXR signalling, as observed in mice (145). Furthermore, inulin administration to high-fat rodents prevents hepatic triglyceride accumulation by modifying gut microbiota by increasing 5-fold the species *Akkermansia muciniphila* (146). However, in a 2024 randomised clinical trial with patients suffering from NAFLD maintaining a stable body weight during the trial, who supplemented with 16g per day of inulin-type fructans, even though the prebiotics increased faecal *Bifidobacterium* bacterial concentration, they did not impact neither hepatic fat accumulation nor inflammatory and hepatic markers (147).

## Synbiotics in NAFLD

Synbiotics are referred to as a mixture comprising live microorganisms (such as probiotics) and substrates (including prebiotics) selectively utilised by host microorganisms that confer a health benefit on the host (148). Oral administration of synbiotics (FOS plus probiotic strains) to patients with NAFLD for 28 weeks in combination with lifestyle modifications is more effective than lifestyle modifications alone in ameliorating NAFLD, especially through further reduction in hepatic and inflammatory markers (149). Supplementation with *Bifidobacterium longum* plus FOS with lifestyle changes (exercise and diet) to NASH is superior to solely lifestyle changes by an additional decrease in aminotransferases (particularly AST) levels, insulin resistance, inflammatory markers, serum endotoxemia and liver steatosis (150). In rodents with NAFLD induced by a high-fructose diet, the addition of FOS plus *Lactobacillus fermentum CECT5716* to their diet improves gut dysbiosis, enhances gut barrier integrity, and thus prevents the development of liver steatosis (151). Furthermore, it seems that the administration of synbiotics to obese children may decrease their body mass index (BMI) and improve their lipid profile (152, 153). In addition, Alves CC, et al. (2017) showed that supplementation synbiotics to rodents increases PPAR-α activity, which upregulates β-oxidation of lipids and decreases lipogenesis by downregulation of SREBP-1c and FAS genes activation and thus ameliorates steatosis (154).

Musazadeh V, et al. (2024) observed in a meta-analysis participating 1,188 patients with NAFLD who were supplemented with synbiotics within 8 to 56 weeks that synbiotics decrease hepatic (AST, ALT and GGT) and inflammatory (CRP and TNF-a) markers as well as improve lipid profile and obesity indicators (155). Liu L, et al. (2019), in a meta-analysis including 782 patients suffering from NAFLD, showed that the administration of probiotics and synbiotics reduces aminotransferases (AST and ALT) and TNF-α levels, ameliorates liver steatosis, decreases liver stiffness and improves lipid profile, although there was not a significant impact on BMI and fasting blood sugar (156). Although microbiome-target therapies (including probiotics, prebiotics and synbiotics) are linked to the amelioration of NAFLD, there is a wide diversity in probiotic strains, dosages and formulations in the literature (129). Supplementation with Lactobacillus paracasei N1115 plus FOS to mice with NAFLD induced improvement in liver steatosis, reduction in TNF-a serum levels and retardation in cirrhosis development (157). However, the administration of synbiotics for 1 year to 24 patients with NAFLD plus significant liver fibrosis (≥F2) did not improve either adipose tissue dysfunction or inflammatory markers, as observed in the 2024 clinical trial (158). In a 2020 clinical trial with 104 patients suffering from NAFLD who supplemented for 1 year with prebiotics (FOS plus *Bifidobacterium animalis subspecies lactis BB-12*), it was shown that there was an alteration in the faecal microbiome, but there was observed a reduction neither in hepatic fat accumulation nor in hepatic fibrosis markers (159). Moreover, in a 2024 meta-analysis including 12,682 individuals with NAFLD who were supplemented with either probiotics, prebiotics or synbiotics, it was shown an overall reduction in aminotransferases (AST and ALT) levels, amelioration of hepatocytes injury indicators, decrease in inflammatory markers (such as TNF-a) and improvement to lipid profile (160).

## FMT in NAFLD

Faecal microbiota transplantation (FMT) is described as the transfer of faecal material containing a minimally manipulated community of microorganisms to a human recipient from a healthy human donor (including autologous transfer), intending a beneficial effect on the recipient’s health through restoration of the gut microbiome (161). For instance, FMT is an effective treatment for recurrent infection from *Clostridioides difficile* (formerly known as *Clostridium difficile*) in immunocompetent individuals (162). There is a rising number of liver diseases (such as hepatic encephalopathy, alcoholic hepatitis and primary sclerosing cholangitis) that FMT may be a potential treatment (34, 163).

Zhou D, et al. (2017) observed that FMT to high-fat diet rodents with NASH induced the mitigation of steatohepatitis through augmentation of beneficial bacteria (such as *Christensenellaceae* and *Lactobacillus*) in the intestinal lumen, increased butyrate faecal levels and enhancement of gut barrier integrity in a mouse model (164). Craven et al. (2020), in a randomised clinical trial (RCT) including 21 participants suffering from NAFLD, noticed that FMT mitigated intestinal permeability after 6 weeks, although it did not impact either hepatic fat accumulation or insulin resistance (165). In another RCT, Witjes JJ, et al. (2020) observed that allogenic FMT from lean vegan donors to obese recipients with NAFLD ameliorates necro-inflammatory histological score and there were some significant changes in the expression of some hepatic genes associated with inflammation and lipid metabolism in comparison with the autologous FMT group with obese NAFLD participants (166). Furthermore, Xue L, et al. (2022), in an RCT with 75 individuals, found that the FMT group had a significant decrease in liver fat accumulation through alterations in gut microbiota, and FMT was more effective in gut microbiota modulation in lean than in obese individuals with NAFLD (167).

## Conclusions

Gut microbiota dysbiosis seems to contribute to NAFLD pathogenesis through multiple pathways (**Table 2**). Emerging data suggests that gut microbiome induces the development of NAFLD mainly through liver inflammation. Since the incidence of NAFLD is increasing worldwide, there is a need for new preventative and therapeutic strategies. Microbiota-target therapies may have a major or supplementary role in NAFLD management in the future. There are some promising data about the administration of probiotics, prebiotics and synbiotics. The future will tell whether the FMT will be indicated for NAFLD prevention or treatment, as it is indicated for recurrent *Clostridium difficile* infection.

**Table 2:** Protective and Risk factors for NAFLD.

Protective Factors Healthy Liver	Risk Factors NAFLD
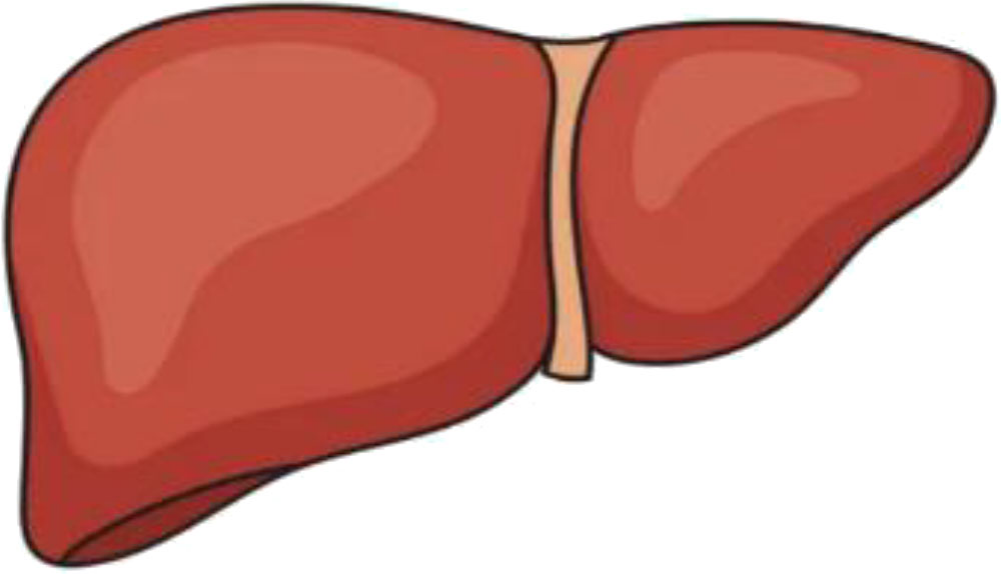	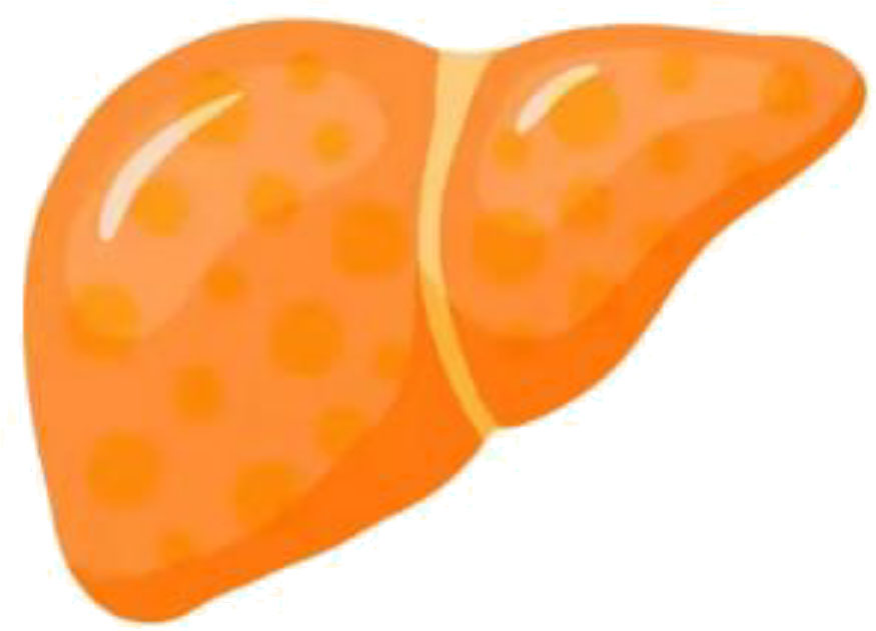
Commensal gut bacteria High gut microbiota diversity Healthy/low-fat diet Normal BMI Exercise ↑Intestinal barrier integrity ↑ SCFAs ↑ Release of GLP-1 ↑ PPAR-α activation Probiotics Prebiotics Synbiotics FMT?	Gut dysbiosis Low gut microbiota diversity Western-type/High-fat diet Obesity/↑ BMI ↓ Intestinal barrier integrity ↑ Bacterial translocation ↑ Release of LPS in systemic circulation ↑ Inflammation in the liver ↑ Hepatocellular injury ↓ SCFAs ↓ Secondary BAs ↓ FXR and TGR5 activation ↑ Ethanol-producing bacteria

↑: increased.

↓: decreased.
